# A Case of Reactive Cervical Lymphadenopathy with Fat Necrosis Impinging on Adjacent Vascular Structures

**DOI:** 10.1155/2016/6019501

**Published:** 2016-10-20

**Authors:** Albert Y. Han, Jacob F. Lentz, Edward C. Kuan, Hiwot H. Araya, Mohammad Kamgar

**Affiliations:** ^1^Department of Medicine, David Geffen School of Medicine at UCLA, Los Angeles, CA 90095, USA; ^2^Medical Scientist Training Program, David Geffen School of Medicine at UCLA, Los Angeles, CA 90095, USA; ^3^Department of Emergency Medicine, David Geffen School of Medicine at UCLA, Los Angeles, CA 90095, USA; ^4^Department of Head and Neck Surgery, David Geffen School of Medicine at UCLA, Los Angeles, CA 90095, USA

## Abstract

A tender neck mass in adults can be a diagnostic challenge due to a wide differential diagnosis, which ranges from reactive lymphadenopathy to malignancy. In this report, we describe a case of a young female with an unusually large and tender reactive lymph node with fat necrosis. The diagnostic imaging findings alone mimicked that of scrofula and malignancy, which prompted a complete workup. Additionally, the enlarged lymph node was compressing the internal jugular vein in the setting of oral contraceptive use by the patient, raising concern for Lemierre's syndrome or internal jugular vein thrombosis. This report shows how, in the appropriate clinical context, and especially with the involvement of adjacent respiratory or neurovascular structures, aggressive diagnostic testing can be indicated.

## 1. Case

A 19-year-old college student presented to a university emergency department with painful swelling on the left side of her neck. The swelling began two weeks earlier. It progressed slowly but had acutely worsened over the previous four days. She did not have a sore throat, but she endorsed worsening odynophagia since four days earlier. She denied any shortness of breath, stridor, or difficulty clearing secretions. She denied fever, skin rash, or axillary/inguinal lymphadenopathy. She recalled no inciting trauma or antecedent symptoms of sickness. She did, however, endorse night chills over the previous week.

The patient's medical history was limited to a diagnosis of streptococcal pharyngitis a month earlier, which had been treated with amoxicillin. Her surgical history included a bilateral tonsillectomy at the age of eight. She had no allergies, and her sole daily medication was an estrogen oral contraceptive pill (OCP). She recalled no family history of hematologic or head and neck malignancy.

The patient was born and raised in California, and she had never traveled outside of the United States or Europe. She had neither risk factors for tuberculosis (TB) nor TB contacts. She had no exposure to pets or animals. She denied drug and tobacco use, although she admitted to regular binge drinking episodes. Notably, the patient's recent strep pharyngitis diagnosis had occurred after a trip to a music festival in the California desert. Several of the friends with whom she had traveled developed similar symptoms, and in each of their cases the final diagnosis was strep pharyngitis.

### 1.1. Physical Exam Findings

The patient's vital signs upon arrival showed a blood pressure of 122/75, a pulse of 84, a temperature of 37.1 degrees Celsius, and respiratory rate of 12. Physical examination revealed a well-developed female in no acute distress. Oropharynx was clear, with no erythema or exudates. She had a nonerythematous, exquisitely tender mass on her left lateral neck over the sternocleidomastoid muscle, measuring approximately four centimeters (cm) in diameter and three cm in height. The skin overlying the neck was intact. The remainder of the exam was unremarkable.

### 1.2. Laboratory Findings

The patient's electrolytes and liver function tests were within normal limits. The complete blood count (CBC) showed an elevated white blood cell count of 12.85, with a neutrophil predominance at 75 percent. The absolute neutrophil count was 9.6, and the absolute lymphocyte count was 2.1. The absolute monocyte count was slightly elevated at 1.1. Her CBC was otherwise unremarkable.

Human immunodeficiency virus (HIV), cytomegalovirus (CMV) IgM, human simplex virus 1/2 IgM, Epstein-Barr virus,* Bartonella henselae*,* Coccidioides*, and* Toxoplasma gondii* serologies were negative. QuantiFERON-TB Gold (Quant-Gold) test was also negative. The lactate dehydrogenase (LDH) level was normal.

### 1.3. Diagnostic Imaging

**Figure 1:**
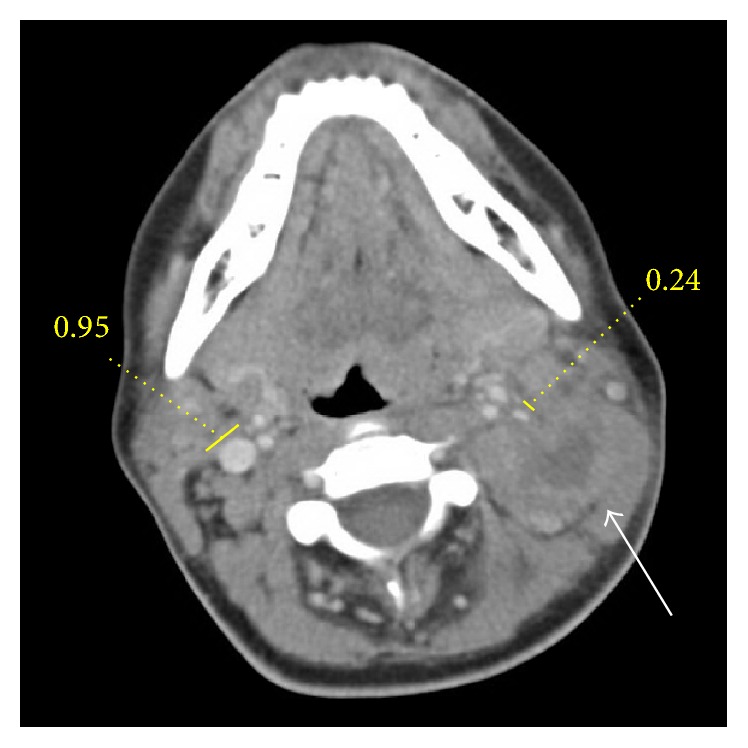
Computed tomography scan of the neck without contrast showing enlarged bilateral lymph nodes. On the patient's left, the largest lymph node (4.1 × 2.7 cm; white arrow) has a central hypodensity (1.9 × 1.2 cm) consistent with fatty necrosis. Additionally, the right internal jugular (IJ) vein measured 0.95 cm in the greatest diameter whereas the left IJ vein was 0.24.

The patient's chest X-ray was unremarkable. A bedside ultrasound of the neck showed findings that were consistent with lymphadenopathy, with the location of the largest lymph node in Level IIB. A contrast-enhanced computed tomography (CT) of the neck revealed multilevel cervical lymphadenopathy bilaterally, with the largest mass measured at 4.1 × 2.7 cm in the left neck. A central hypodense area of this node measured 1.9 × 1.2 cm. The left internal jugular (IJ) vein remained patent but was decreased in caliber secondary to mass effect from the largest node (Figure 1). The diameter of the IJ vein was 0.95 cm on the right and 0.27 cm on the left. Radiology read this as a likely necrotic lymph node. (Figure 1).

Due to the impressive presentation, hypercoagulability (OCP use), venostasis (changes in IJ caliber), and concern for airway compromise, the patient was admitted for expedited evaluation and treatment. Furthermore, as a delayed diagnosis of head and neck cancer is associated with lower survival, otolaryngology was consulted immediately [1].

### 1.4. Hospital Course

The patient's airway remained patent and required no immediate intervention. Given the high suspicion for infection, the patient was started on empiric ampicillin-sulbactam. The swelling began to decrease over the course of treatment, and the patient's leukocyte count downtrended and normalized three days into treatment.

**Figure 2:**
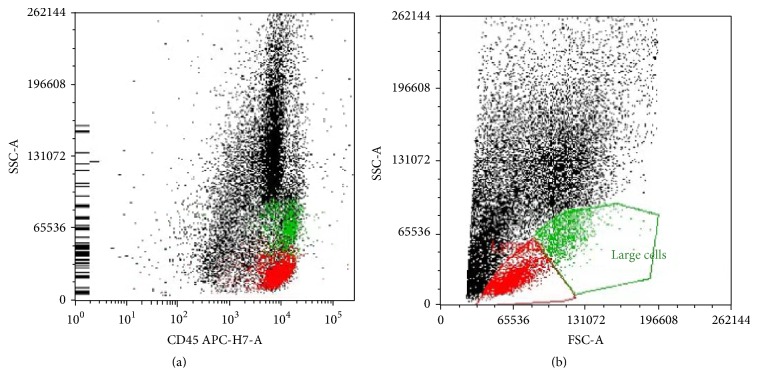
Multiparametric flow cytometry results. CD45+ lymphocyte gate (low SSC-A) was 14% of the total population (a). The lymphocytes were predominantly T cells (12% of total cells; (b)). (a) CD45 versus SSC and (b) FSC versus SSC.

On hospital day 2, the patient underwent ultrasound-guided core needle biopsy. Tissue pathology showed fibroadipose tissue with acute inflammation and fat necrosis. A gram stain and acid-fast bacilli (AFB) stain of the specimen were negative, as was the gomori methenamine silver stain. The bacterial and fungal cultures of the aspirate were subsequently negative. Cytology revealed abundant neutrophils in a background of histiocytes and occasional scattered lymphocytes. Flow cytometry was negative for monoclonal proliferation (Figure 2).

The patient continued to improve. However, due to the diagnostic uncertainty from the core needle specimen, an excisional biopsy was performed on hospital day 4. The final pathology showed a mixture of T and B cells and irregular fragments of lymphoid cortex without light chain restriction, granulomas, or other focal lesions. These findings were consistent with reactive lymphoid tissue. The size and tenderness of the lymph node continued to decrease, and the patient was discharged with return precautions and outpatient follow-up on hospital day 6.

### 1.5. Diagnosis

 The diagnosis was a reactive lymph node.

## 2. Discussion

A tender neck mass is a common presenting symptom in the adult population [2, 3]. The evaluation of a neck mass begins with a careful history related to the lesion (i.e., location, migration, temporal course, and associated symptoms). Patient-specific risk factors such as previous trauma, relevant travel, animal contact, and past medical history should be reviewed [2, 3]. Physical exam should not only include a characterization of the lesion but also an assessment of cranial nerve integrity and function. The security of the airway is crucial and must be checked during the initial assessment. Any sign of possible impending respiratory compromise warrants admission [4]. Possible odontogenic etiology, such as dental caries, must be considered, as complications from infection can negatively impact the course of treatment [3].

As a general rule, swollen nodes other than supraclavicular/Level V nodes usually result from reactive lymphadenopathy or infectious/viral lymphadenitis [5–7]. In the present case, a moderate leukocytosis and normal LDH lowered the suspicion of malignancy. However, a possible lymphatic spread of occult primary cancer remained on the differential, especially as the Level IIB nodes drain the oropharynx and the nasopharynx [8]. In this patient, the largest lymph node was in Level IIB, with a maximum diameter of 4.1 cm. The commonly accepted radiological criteria for lymph node malignancy is a ratio between the dimension of the long and short axes less than 2 [6, 9]. In the present case, the ratio was 1.52 with evidence of a necrotic core, and this prompted further investigation. Of note, CT of the neck is sensitive but not specific in differentiating an inflamed lymph node or abscess from metastatic cancer, as they often appear identical on imaging [2].

Tuberculosis cervical lymphadenitis (i.e., scrofula or King's evil) is perhaps the most famous etiology of a neck mass. Scrofula most commonly presents in young patients as large painless lymph nodes [10, 11]. Interestingly, only one-third of scrofula patients have a documented history of TB [12, 13]. The salient features of scrofula on a CT scan include central necrosis, nodal matting, and minimal periadenitis [14]. The gold standard test for diagnosing scrofula is tissue sampling and AFB stain and culture. However, the newer Quant-Gold test detects the release of interferon-*γ* by leukocytes, which are sensitized after incubation with synthetic peptides similar to* Mycobacterium tuberculosis* proteins. The Quant-Gold test can also detect nontuberculous mycobacteria, as identical proteins are also found in the species* Mycobacterium kansasii, Mycobacterium szulgai*, and* Mycobacterium marinum* [15, 16]. Although the patient in this case had a painful neck mass and a negative AFB stain and culture, it would have been critical to rule out pulmonary involvement if a diagnosis of scrofula had been made, as the public health implications for this are significant [11].

Other rare causes of neck masses include Kikuchi disease, Castleman disease, Kimura disease, and Rosai-Dorfman disease. Among these, Kikuchi disease was placed on the differential because it typically presents as cervical lymphadenopathy in a young woman associated with fever and constitutional symptoms [17]. Diagnosis is made by a tissue lymph node histology demonstrating paracortical areas of necrosis with the proliferation of histiocytes in the absence of neutrophils [18]. The natural history is benign and ultimately self-limiting [18]. The tissue analysis of the excisional biopsy in this case showed abundant neutrophils; this eliminated Kikuchi disease from the differential.

Bacterial infection can cause lymphadenopathy via an activated inflammatory response or direct hematogenous spread of bacteria into nodes. Reactive lymphadenopathy also emerges with the stimulation of the immune system by regional infectious processes, such as upper respiratory infections, stomatitis, or dental caries [14, 19]. Many viral infections, including HIV, infectious mononucleosis, and CMV, can result in significant cervical lymph node swelling via this mechanism. Bacteria from an odontogenic or salivary infection can directly travel to the cervical nodes, leading to frank abscess formation [2]. Patients with bacterial abscesses have pain, swelling, and erythema and possibly present with a fever. The bacterial abscess considered in this case was cat scratch disease, which is caused by the bacteria* Bartonella henselae* and is often found in patients younger than 21 years old with exquisitely tender lymph nodes [19, 20]. IgM/IgG serology is the diagnostic test of choice for* B. henselae* infection. In the present case, the serology was negative.

Poorly controlled odontogenic or pharyngeal infections can result in vascular complications. The patient in this case had at least two elements of Virchow's triad: hypercoagulation (OCP use) and stasis of blood flow (reduced caliber of IJ by 25%). Vascular complications are uncommon, but they include IJ venous thrombosis (IJVT) and thrombophlebitis of the IJ vein (Lemierre's syndrome). IJVT most commonly occurs in the setting of central line infection or tumor invasion into the vasculature [21, 22]. Lemierre's syndrome is characterized by a triad of recent oropharyngeal infection, IJVT identified on CT, and confirmed anaerobic bacterial infection (most commonly* Fusobacterium necrophorum*) [23]. Approximately 30% of the patients experience a preceding upper respiratory infection. Upon first presentation, 23% of patients display a neck mass and approximately 20% complain of neck pain [24]. Although rare, the patient in this case had a recent history of pharyngitis and was at an increased risk for thrombosis due to her OCP use; she was therefore monitored closely during hospitalization.

It was ultimately concluded that an occult chronic bacterial infection was the cause of the patient's lymphadenopathy. It was hypothesized that her history of tonsillectomy left her oropharynx more prone to infection. Due to the degree of swelling and the significant compression of the left IJ in the setting of a hypercoagulable state, admission and extensive investigation were clinically warranted. Although neither this complication nor respiratory compromise developed, and while all stains, cultures, and serologies were negative, this case serves as a reminder that reactive lymph nodes can mimic many features of classic infectious and neoplastic processes, can grow to a formidable size, and can compress critically important adjacent structures.
